# The tryptanthrin derivative g2 suppresses tongue squamous cell carcinoma via PI3K/AKT/P53 cathway modulation: evidence from *in vitro* and *in vivo* studies

**DOI:** 10.3389/fphar.2026.1789002

**Published:** 2026-06-18

**Authors:** Jing Miao, Shijie Zhang, Mengxiang Wang, Zeng Li, Na Lv, Ming Sun

**Affiliations:** 1 Department of Stomatology, The First Affiliated Hospital of Anhui Medical University, Anhui Medical University, Hefei, China; 2 Inflammation and Immune Mediated Diseases Laboratory of Anhui Province, School of Pharmacy, Anhui Medical University, Hefei, China

**Keywords:** anti-tumor, G2, PI3K/Akt/P53 pathway, tryptanthrin derivatives, TSCC

## Abstract

**Introduction:**

Tongue squamous cell carcinoma (TSCC) is a prevalent and aggressive oral malignancy with a poor prognosis and limited treatment options, underscoring the need for novel therapeutic agents. Tryptanthrin derivatives have shown promising anticancer potential across various cancers. This study aimed to evaluate the antitumor efficacy and elucidate the mechanism of action of a novel tryptanthrin derivative, **g2**, against TSCC.

**Methods:**

The cytotoxic effect of **g2** was initially screened against CAL-27 and SCC9 TSCC cells using a CCK-8 assay among 36 derivatives. Its half-maximal inhibitory concentration (IC_50_) was determined. A comprehensive set of *in vitro* assays, including scratch wound-healing, Transwell migration, flow cytometry for apoptosis and cell cycle analysis, transcriptome sequencing, and western blotting, were employed to assess its effects on cell migration, apoptosis, cycle progression, and underlying signaling pathways. For *in vivo* validation, a xenograft model was established by implanting CAL-27 cells into nude mice, which were then treated with **g2** (10 or 20 mg/kg) via intratumoral injection. Tumor growth was monitored, and harvested tissues were analyzed by western blotting.

**Results and Discussion:**

**g2** demonstrated the most potent cytotoxicity, with IC_50_ values of 1.997 μM for CAL-27 and 2.134 μM for SCC9 cells. It significantly inhibited cell migration and invasion, induced apoptosis, and caused S-phase cell cycle arrest in a dose-dependent manner. Transcriptome sequencing and western blotting analyses revealed that **g2** activated the PI3K/AKT/P53 signaling pathway, evidenced by the downregulation of PI3K/AKT and upregulation of P53. *In vivo*, **g2** treatment markedly suppressed tumor growth without observable systemic toxicity, and consistent modulation of the PI3K/AKT/P53 pathway was confirmed in tumor tissues. These findings collectively demonstrate that the tryptanthrin derivative **g2** exerts significant anti-TSCC effects both *in vitro* and *in vivo*, primarily through the modulation of the PI3K/AKT/P53 pathway, highlighting its potential as a promising therapeutic candidate. Further studies are warranted to explore its full mechanistic spectrum and efficacy in broader models.

## 1 Introduction

Among the types of cancer, the second leading cause of death globally, head and neck squamous cell carcinoma (HNSCC) accounts for approximately 500,000 new cases each year (Ba et al., 2020; Wang et al., 2023; Zheng et al., 2024). Among the types of HNSCC, tongue squamous cell carcinoma (TSCC) is the most common oral cancer in China, and it is highly prone to spreading to lymph nodes in the head and neck region (Lu et al., 2021). Although surgery remains the primary treatment for TSCC, it often leads to significant challenges (Muñoz-Grez et al., 2025), including impaired speech, difficulty eating and breathing, and facial deformities, and it has a 5-year survival rate of less than 50% (Duan et al., 2022; Freitas et al., 2020). The tongue’s constant movement, rich blood supply, and extensive lymphatic network contribute to a high rate of recurrence, further worsening the prognosis (Li et al., 2021; Sur et al., 2019). Efforts to improve outcomes have been hindered by a limited understanding of the molecular mechanisms driving TSCC. Existing treatments, such as cisplatin and fluorouracil, typically provide only modest benefits, as they lack specificity, and are associated with severe side effects (Patil et al., 2023), including bone marrow suppression, underscoring the urgent need for more effective and targeted approaches (Florido et al., 2022; Wang et al., 2021).

Indigo plants, particularly those from the genus Strobilanthes cusia, have long been valued in Asian cultures as natural sources of dyes for the textile and printing industries (Catanzaro et al., 2020; Yu et al., 2010). First isolated and purified by Honda’s research team, tryptanthrin has become the focus of biomedical research because of its therapeutic potential. Recent studies have highlighted tryptanthrin’s promising applications in anticancer (Gao et al., 2021; Jun et al., 2015; Shabna et al., 2022; Zou et al., 2023), anti-inflammatory (Kirpotina et al., 2020; Yu et al., 2007), and antifungal therapies (Sulaiman et al., 2022; Long et al., 2023; Sudheendran Leena et al., 2022). In cancer research, tryptanthrin has demonstrated significant efficacy against various cancers, such as liver and lung cancers, by inhibiting cell proliferation, migration, and metastasis through interference with cell growth cycles (Chang et al., 2024; Zeng et al., 2021; Liao and Leung, 2013; Lin et al., 2023). Its strong time- and dose-dependent inhibitory effects on breast cancer cells further underscore its potential as an effective anticancer agent. Additionally, tryptanthrin has been shown to influence key molecular targets, such as modulating the MDR1 gene promoter to potentially reduce P-gp production and lowering mutant P53 protein levels. These findings suggest that tryptanthrin is a versatile compound with considerable promise for cancer therapy (Yang et al., 2013).

**FIGURE 1:**
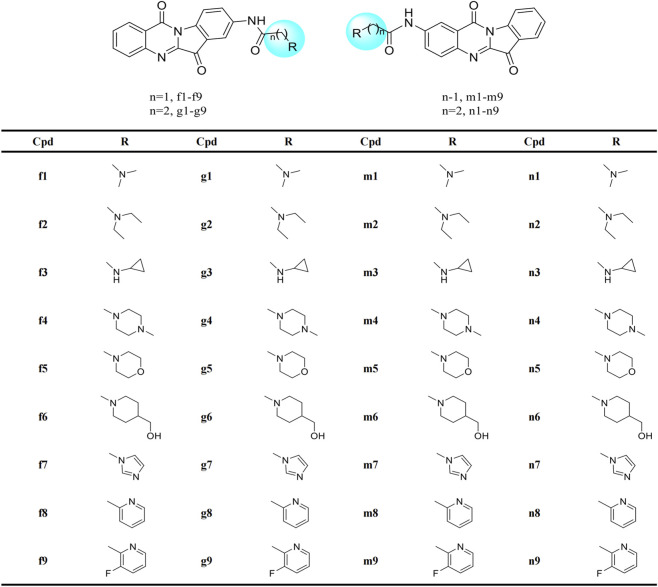
Structural formula of tryptanthrin derivatives.

Based on the aforementioned theoretical framework, our research team designed and synthesized a series of tryptanthrin derivatives (Figure 1). The synthesized tryptanthrin derivatives exhibited high purity (>95%) as determined by HPLC analysis. When the derivatives were subsequently evaluated for their efficacy against TSCC (Xia et al., 2024), the compound **g2** demonstrated the strongest antitumor activity, significantly reducing the viability of CAL-27 and SCC9 cells, two TSCC cell lines, inhibiting their migration and invasion, and promoting apoptosis. Transcriptome sequencing further revealed that **g2** activates the PI3K/AKT/P53 signaling pathway, highlighting potential new gene targets. When animal models were used to evaluate the *in vivo* efficacy of **g2**, the results confirmed its ability to inhibit tumor growth, providing further evidence of its potential as a therapeutic agent for TSCC.

## 2 Materials and methods

### 2.1 Materials

Thirty-six tryptanthrin derivatives were provided by our research group, which is affiliated with the School of Pharmacy at Anhui Medical University. These derivatives were dissolved in dimethyl sulfoxide (DMSO) sourced from Solarbio (Beijing, China). A stock solution of the tryptanthrin derivatives was prepared in DMSO at a concentration of 10 mM and stored at −20 °C for future use. After being thawed, the stock solution was maintained at 4 °C in a light-protected environment.

### 2.2 Cell culture and reagents

CAL-27 and SCC9 cell lines were provided by Anhui Medical University. Both CAL-27 and SCC9 cells were cultured in high-glucose Dulbecco’s modified Eagle’s medium (DMEM, Thermo Fisher Scientific, United States) supplemented with 10% fetal bovine serum (FBS; Biological Industries, Israel) and 100 U/mL penicillin/streptomycin. For SCC9 cells, the medium was further supplemented with 400 ng/mL hydrocortisone (Beyotime, Shanghai, China). The cells were maintained at 37 °C in an incubator containing humidified air with 5% CO_2_ according to standard procedures. To ensure cellular vitality, the cultures were passaged no more than 3 months after initial resuscitation.

### 2.3 Cell viability analysis

Cells were seeded at 1 × 10^4^ cells/well in 96-well plates with growth media containing 10% FBS and incubated overnight at 37 °C. On the second day, cells were treated with each of the 36 tryptanthrin compounds at a concentration of 5 μM. The compounds were dissolved in DMSO and then diluted with culture medium to achieve the desired final concentrations. In all treatment groups and the vehicle control group, the final concentration of DMSO was maintained at 0.1% (v/v). Control wells received an equivalent amount of DMSO (0.1%) without any compound. After pretreatment, Cell Counting Kit-8 assay (CCK-8) was performed by adding 10 μL of CCK-8 solution (Beyotime, Shanghai, China) to the cells and incubating them for 2 h at 37 °C in the dark. Absorbance was measured at 492 nm, and the experiment was repeated three times. The cell viability was calculated as the ratio of the mean optical density (OD) value between cells treated differently in each group. The percentage of viable cells was determined using the following formula: ratio (%) = (OD [treat] – OD [Blank])/(OD [Control] – OD [Blank]) ×100%. The half-maximal inhibitory concentration (IC_50_) was determined as the concentration resulting in 50% cell growth reduction compared with the untreated cells. IC_50_ values were calculated by dose-response curve fitting using GraphPad Prism 10 software.

### 2.4 Scratch wound-healing assay

For the scratch assay, CAL-27 and SCC9 cells were inoculated in six-well plates with 1 × 10^5^ cells per well and allowed to grow overnight. On the second day, the cell layer was wounded by scratching with a sterilized yellow pipette tip. Subsequently, the cells were treated with DMSO (2 μM), the positive drug cisplatin (2 μM) (Beyotime, Shanghai, China), and serial dilutions of **g2** (0, 1, 2, and 5 μM) for 24 h. Images were captured under a digital single lens reflex camera (Leica, Wetzlar, Germany) at 24 h. The healing of the wounds through cell migration was quantified by measuring the wound distance.

### 2.5 Transwell migration assay

Transwell migration assay was performed using 24-well cell culture inserts (8-μm pore; Corning, Corning, NY, United States) without chambers matrigel. Briefly, 5 × 10^4^ cells were grown in the upper chamber of a Transwell apparatus (membrane pore size = 8 μm) before 200 μL of serum-free DMEM (high glucose) was added to the upper chamber. Next, 24-well plates were supplemented with DMEM high-glucose medium containing 20% FBS and compound **g2** (0, 1, 2, or 5 μM). After allowing the cells to migrate for 24 h, the cells in the upper surface of the membrane were removed using a cotton swab, and the membranes were fixed with methanol and stained with crystal violet. The number of migrating cells was determined by averaging cell counts from nine randomly selected fields (×100 magnification). Each experiment was repeated three times.

### 2.6 Quantification of apoptosis by flow cytometry

The quantification of apoptotic CAL-27 and SCC9 cells was conducted utilizing the Annexin V-fluorescein isothiocyanate (FITC) apoptosis detection kit (Invitrogen, Carlsbad, CA, United States). Specifically, CAL-27 and SCC9 cells were exposed to **g2** at concentrations of 0, 1, 2, and 5 μM for 24 h. Subsequently, the cells were harvested and subjected to three washes with cold phosphate-buffered saline (PBS). The cell pellets were then resuspended in Annexin V-labeling solution and incubated at an ambient temperature for 15 min. For dual staining, the cells were treated with 5 μg/mL of propidium iodide (PI), followed by the addition of 400 μL of 1×Binding buffer to each experimental group. After thorough mixing, the samples were maintained on ice and promptly analyzed using a Coulter Epics XL flow cytometer (Beckman Coulter, Miami, FL, United States).

### 2.7 Cell cycle analysis

CAL-27 and SCC9 cells were exposed to varying concentrations of **g2** (0, 1, 2, or 5 μM) for 24 h. Following treatment, the cells were harvested and fixed in 70% ice-cold ethanol overnight. Subsequently, the cells were treated with 500 μL of a PI/RNase A working solution comprising 50 μg/mL of PI (P4170, Sigma Aldrich, St. Louis, MO, United States) and 100 μg/mL of RNase A (10109142001, Roche Diagnostics, Mannheim, Germany). Cell cycle distribution was then analyzed via flow cytometry. Additionally, apoptosis was assessed using the PI/Annexin V-FITC kit (556547, BD Biosciences, Franklin Lakes, NJ, United States), followed by flow cytometric evaluation to determine cell cycle progression and apoptotic status.

### 2.8 Transcriptome sequencing

CAL-27 and SCC9 cells (5 × 10^6^ cells) were cultured in T25 breathable cell flasks and pretreated for 24 h with compound **g2** (2 μM). TRIzol Reagent (1 mL) was added to pretreated cells and untreated cells, fully lysed, and frozen for storage. Total RNA was extracted from cells/tissues using TRIzol reagent (Invitrogen, United States) according to the manufacturer’s protocol. RNA purity and concentration were assessed using a NanoDrop 2000 spectrophotometer (Thermo Scientific, United States), and RNA integrity was evaluated using the Agilent 2,100 Bioanalyzer (Agilent Technologies, United States). Briefly, mRNA was enriched using oligo (dT) beads, fragmented, and reverse-transcribed into cDNA. The libraries were sequenced on the Illumina NovaSeq 6,000 platform, generating 150 bp paired-end reads.

The raw sequencing data (FASTQ files) were processed and analyzed as follows. Quality control was performed by removing adapter sequences and low-quality reads using fastQC. The clean reads were then aligned to the human reference genome Ensembl_v107 using HISAT2 with default parameters. Gene expression levels were quantified as FPKM using StringTie (Lyu et al., 2023).

Differential expression analysis between treatment and control was performed using DESeq2. Genes with an absolute log2 fold change (|log2FC|) > 1 and a false discovery rate (FDR) < 0.05 were considered significantly differentially expressed. To explore the biological functions of the differentially expressed genes (DEGs), Gene Ontology (GO) enrichment analysis and Kyoto Encyclopedia of Genes and Genomes (KEGG) pathway enrichment analysis was performed using the OmicStudio tools (available at: https://www.omicstudio.cn/tool). Terms with a corrected p-value <0.05 were considered significantly enriched.

### 2.9 Western blotting

CAL-27 and SCC-9 cells were seeded in six-well plates at a density of 5 × 10^5^ cells per well. The experimental groups were subjected to **g2** treatment (0, 1, 2, and 5 μM) for 24 h, after which the cells were lysed on ice for 30 min to extract proteins. The resulting cell lysates were subjected to sodium dodecyl sulfate–polyacrylamide gel electrophoresis (SDS-PAGE). Equal quantities of protein (50 μg) were resolved on 12% SDS-PAGE gels and subsequently transferred onto polyvinylidene fluoride (PVDF) membranes (Millipore, United States). The membranes were blocked with 5% non-fat milk for 2 h at room temperature to prevent non-specific binding, followed by overnight incubation at 4 °C with specific primary antibodies. Primary antibodies targeting phospho-PI3K (1:1500, AF3242), total PI3K (1:1500, AF6242), phospho-AKT (1:1500, AF0016), total AKT (1:1500, AF6261), P53 (1:1500, AF0879), and GAPDH (1:1500, AF7021) were procured from Affinity (United States), with GAPDH serving as the internal loading control. After primary antibody incubation, the membranes were exposed to horseradish peroxidase-conjugated anti-rabbit or anti-mouse secondary antibodies for 2 h at room temperature. Protein bands were visualized using an enhanced chemiluminescence detection kit (WBKLS0500, Millipore, Burlington, MA, United States). Quantitative analysis of the Western blotting bands was performed using ImageJ software, where band intensity (area × optical density) was measured for each experimental condition. This approach ensured accurate normalization and comparison of protein expression levels across groups.

### 2.10 *In vivo* experiments

#### 2.10.1 Experimental animals

30 female BALB/c nude mice (4 weeks; 14–18 g; Forbes Biotechnology Co., Ltd.) were housed in a room at 28 °C, relative humidity of 40%–60%, and exposed to a light–dark cycle of approximately 12 h. The mice had free access to chow and water. There mice were randomly divided into control and **g2** (10 mg/kg) and **g2** (20 mg/kg) groups. Each group have 10 mice. This experimental protocol adhered to animal care standards approved by the Ministry of Science and Technology of the People’s Republic of China and complied with the “Animal Research: Reporting of *In Vivo* Experiments” guidelines, and were approved by the Animal Care and Use Committee of Anhui Meical University (NO.20201111).

#### 2.10.2 Drug treatment

CAL-27 cells were orthotopically implanted into the right axillary region of nude mice in the experimental group. When the tumor grew to 5 mm × 5 mm, compound **g2** at a dose of 10 or 20 mg/kg (Supplementary Table S1) was administered by intratumoral injection. Tumor size was measured every 5 days, and body weight was measured every 7 days. The control group received no drug treatment. After 35 days post-inoculation, all mice were humanely euthanized. Euthanasia was performed by first inducing deep anesthesia with 5% isoflurane in an induction chamber, followed by confirmation of death via cervical dislocation.

#### 2.10.3 Tumor tissue immunoblotting

The experimental protocol commenced with the precise dissection of tumor specimens under cryogenic conditions, utilizing instruments that had been pre-cooled to maintain tissue integrity. Each tissue aliquot was subjected to mechanical disruption and homogenized in the presence of 200 μL of ice-cold lysis buffer. The homogenate was then agitated intensively for 10 min employing a mechanical homogenizer, followed by centrifugal separation at 12,000 revolutions per minute for 15 min to facilitate the isolation of the soluble fraction. The acquired supernatant was meticulously aliquoted and preserved at a temperature of −20 °C to ensure protein stability. Subsequent to protein extraction, the samples were resolved and subsequently transferred onto PVDF membranes to immobilize the proteins. These membranes underwent a tripartite washing regimen with Tris-buffered saline supplemented with Tween-20 to remove non-specifically bound materials. Following washing, the membranes were incubated with a panel of primary antibodies, including monoclonal antibodies specific to phosphorylated and total PI3K, as well as polyclonal antibodies targeting phosphorylated AKT, total AKT, and P53, at 4 °C overnight to ensure optimal antibody–antigen interactions. After incubation, the membranes were exposed to species-matched secondary antibodies for 2 h to enable primary antibody complex detection. The immunoblots were then processed to visualize protein bands, permitting qualitative and quantitative evaluation of target proteins. This method ensured rigorous and reproducible analysis of tumor tissue protein expression profiles.

### 2.11 Statistical analysis

Data are presented as the mean ± standard deviation (SD) from a minimum of three independent experiments. Statistical significance was determined by one-way analysis of variance, with **P < 0.01 considered statistically significant. All analyses were conducted using GraphPad Prism 10.0.

## 3 Results

### 3.1 Toxicity study of tryptanthrin derivatives on CAL-27 and SCC9 cells

**FIGURE 2:**
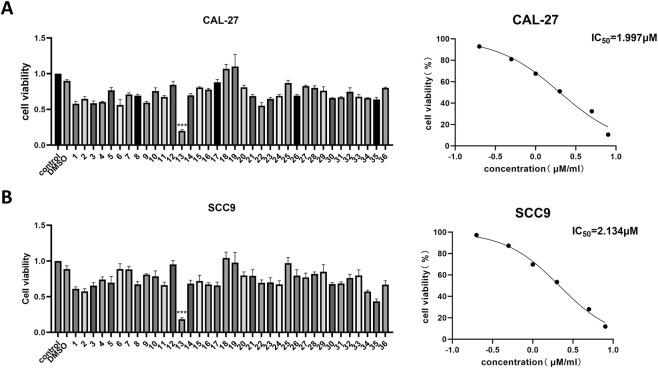
**(A,B)** A CCK8 cytotoxicity experiment was conducted to measure the cytotoxicity of 36 tryptanthrin derivatives and DMSO at a concentration of 5 μM. The control group received no drug treatment. Compound **g2** showed strong cytotoxicity, ***P < 0.001. CAL-27 and SCC9 cells were treated with six doses of the initially screened drug **g2**. The IC_50_ values were 1.997 μM for CAL-27 cells and 2.134 μM for SCC9 cells.

Cell viability was evaluated using CCK-8 assay. CAL-27 and SCC-9 cells were treated with 36 distinct tryptanthrin derivatives at a uniform concentration of 5 μM for 24 h. A control group was treated with DMSO. Among the tested compounds, the 13th derivative, designated as **g2**, demonstrated the most potent cytotoxic activity against the TSCC cells. Based on these findings, **g2** was selected for subsequent investigations. To determine the IC_50_ concentration, the cells were exposed to a range of **g2** concentrations (0, 0.5, 1, 2, 5, and 8 μM). The calculated IC_50_ values were 1.997 μM for the CAL-27 cells and 2.134 μM for the SCC-9 cells (Figure 2), indicating the compound’s efficacy in inhibiting cell proliferation. These results underscore the potential of **g2** as a promising therapeutic agent for further exploration in TSCC research.

### 3.2 Compound **g2** inhibits the migration of CAL-27 and SCC9 cells

**FIGURE 3:**
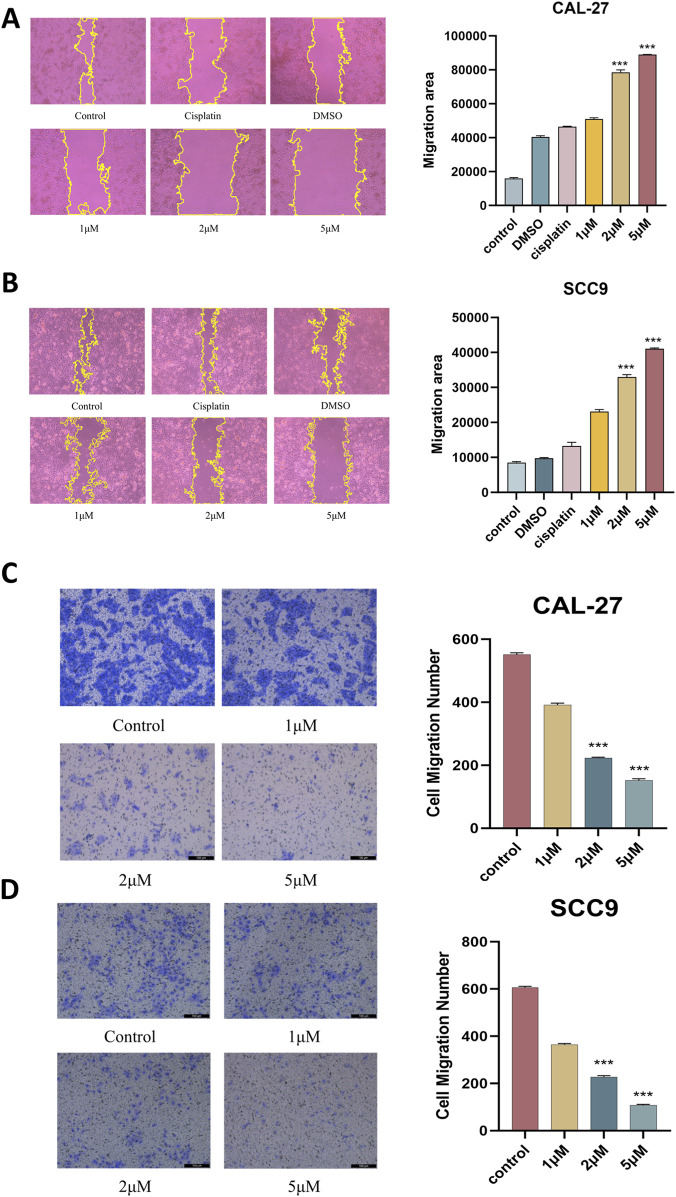
**(A,B) g2** inhibited the migration of CAL-27 and SCC9 cells *in vitro*. No drugs/compounds (control group), DMSO (2 μM), the positive drug cisplatin (2 μM) (Sa et al., 2024), and **g2** (0, 1, 2, and 5 μM) were used to treat CAL-27 and SCC9 cells for 24 h. A scratch healing experiment showed that **g2** inhibited the scratch healing ability of human tongue squamous cell carcinoma cells and that this ability increased with an increase in concentration. The healing areas of the CAL-27 and SCC9 cell scratches were statistically analyzed. ***P < 0.001. **(C,D)** Transwell The migration and invasion of CAL-27 and SCC9 cells were detected after 24 h of control treatment or treatment with **g2** (1, 2, and 5 μM). The number of migrated cells was statistically analyzed. ***P < 0.001.

The inhibitory capacity of **g2** on the migration and invasion of CAL-27 and SCC9 cells was next examined. Cell scratch experiments revealed that the addition of compound **g2** significantly increased the healing rate of cell scratches compared with the addition of no treatment, DMSO, or cisplatin. As the dosage of **g2** increased, the healing rate continued to increase. As can be inferred from the image data, in the experiments conducted on CAL-27 cells, the wound closure area in the control group was below 20,000 units. In contrast, the wound closure areas in the DMSO, cisplatin, and **g2** (1 μM) group exhibited a gradual increase, maintaining a range between 40,000 and 60,000 units. At a **g2** concentration of 2 μM, the scratch wound closure area attained 80,000 units. When the concentration was increased to 5 μM, the wound closure area reached its peak, approaching 100,000 units. A similar trend was observed in the experiments involving SCC9 cells; the wound closure areas were the largest in the **g2** (2 μM and 5 μM) groups, with the 5 μM group approaching nearly 100,000 units. Additionally, following 24 h of **g2** treatment, the cell count in the lower compartment of the Transwell chamber was notably reduced compared with that of the control group. These results indicate that **g2** inhibits the migration and invasion of CAL-27 and SCC9 cells, with the inhibitory effect intensifying as the compound dosage increases. The results therefore demonstrate that the tryptanthrin derivative **g2** exhibits a potent inhibitory effect on the migration and invasion of CAL-27 and SCC9 cells (Figure 3).

To investigate the molecular mechanisms underlying the reduced migration capacity, we examined the expression of key EMT markers by [RNA-seq/qPCR] (Supplementary Figure S2), treatment with **g2** significantly upregulated the epithelial marker E-cadherin (CDH1) in CAL-27 and SCC9 cells, while the mesenchymal markers Vimentin (VIM) and N-cadherin (CDH2) showed minimal changes. These results suggest that **g2** promotes an epithelial phenotype, which is consistent with the observed suppression of cell migration.

### 3.3 Tryptanthrin derivative **g2** induces apoptosis of human TSCC CAL-27 and SCC9 cells

**FIGURE 4:**
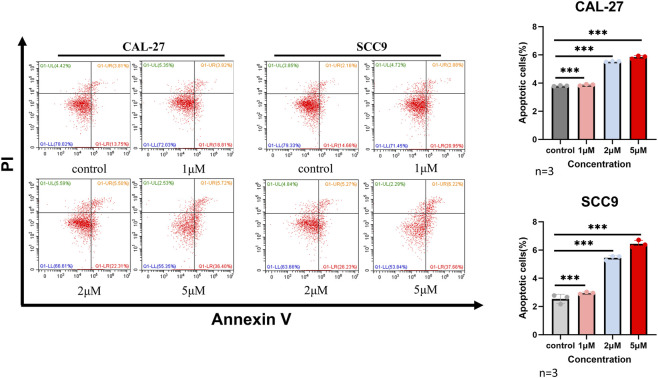
Effect of **g2** on apoptosis in CAL-27 and SCC9 cells. Cells were treated with increasing concentrations of **g2** (1, 2, and 5 μM) for 24 h. Apoptosis was assessed by Annexin V/PI double staining using flow cytometry. **(A)** Representative flow cytometry plots of CAL-27 and SCC9 cells. The lower-right quadrant (Q1-LR) indicates early apoptotic cells (Annexin V^+^/PI^−^), and the upper-right quadrant (Q2-UR) indicates late apoptotic/necrotic cells (Annexin V^+^/PI^+^). Numbers represent the percentage of cells in each quadrant. **(B)** Quantification of total apoptotic cells (early + late) from three independent biological replicates (n = 3 per group). Data are shown as mean ± SD. *p < 0.05, **p < 0.01, ***p < 0.001 vs. control group.

The Annexin FITC apoptosis detection kit was employed to assess the apoptotic effects of the tryptanthrin derivative **g2** on CAL-27 and SCC-9 cells following 24 h of exposure to a gradient concentration range. Quantitative analysis of apoptosis induction in TSCC cells using flow cytometry demonstrated that **g2** treatment significantly increased the proportion of apoptotic cells in both CAL-27 and SCC-9 cells in a dose-dependent manner. Specifically, in CAL-27 cells, the apoptotic rates were 3.82%, 5.50%, and 5.72% for the 1, 2, and 5 μM treatment groups, respectively. Similarly, in SCC-9 cells, the apoptotic rates were 2.88%, 5.27%, and 6.22%, respectively, for the corresponding concentrations (Figure 4).

### 3.4 Compound **g2** arrests CAL-27 and SCC9 cells in the S phase

**FIGURE 5:**
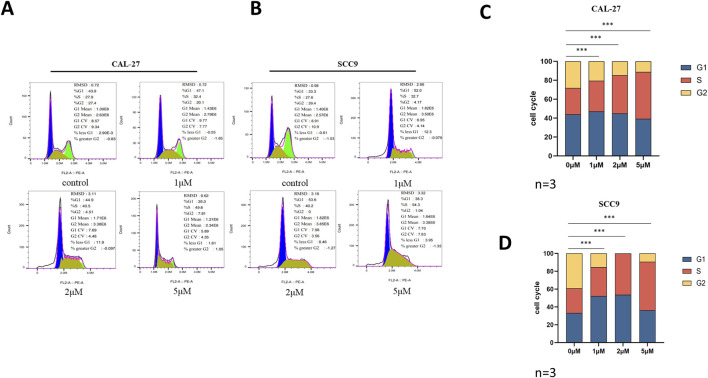
Cell cycle analysis of CAL-27 and SCC9 cells treated with **g2**. **(A,B)** Representative DNA content histograms from one of three independent experiments. **(C,D)** Quantification of the percentage of cells in G0/G1, S, and G2/M phases, respectively, from three independent biological replicates (n = 3 per group). Data are mean ± SD. ***p < 0.001 vs. control.

Following treatment with varying concentrations of the tryptanthrin derivative **g2** (0, 1, 2, and 5 μM), flow cytometric analysis was conducted to evaluate alterations in cell cycle distribution. The results demonstrated a dose-dependent modulation of the cell cycle in treated cells compared with control cells. Notably, a significant reduction in the proportion of cells associated with the G1-S transition phase was observed as the concentration of **g2** increased. Concurrently, a marked accumulation of cells in the S phase was detected, indicative of S phase arrest. At a concentration of 2 μM, SCC-9 cells exhibited complete arrest in the S phase, with no progression to subsequent phases of the cell cycle. These findings suggest that **g2** exerts a potent inhibitory effect on cell cycle progression, particularly by disrupting G1-S transition and inducing S phase arrest, which may contribute to its anti-proliferative activity in TSCC cells (Figure 5).

### 3.5 RNA sequencing shows that **g2** can activate the PI3K/AKT/P53 pathway

**FIGURE 6:**
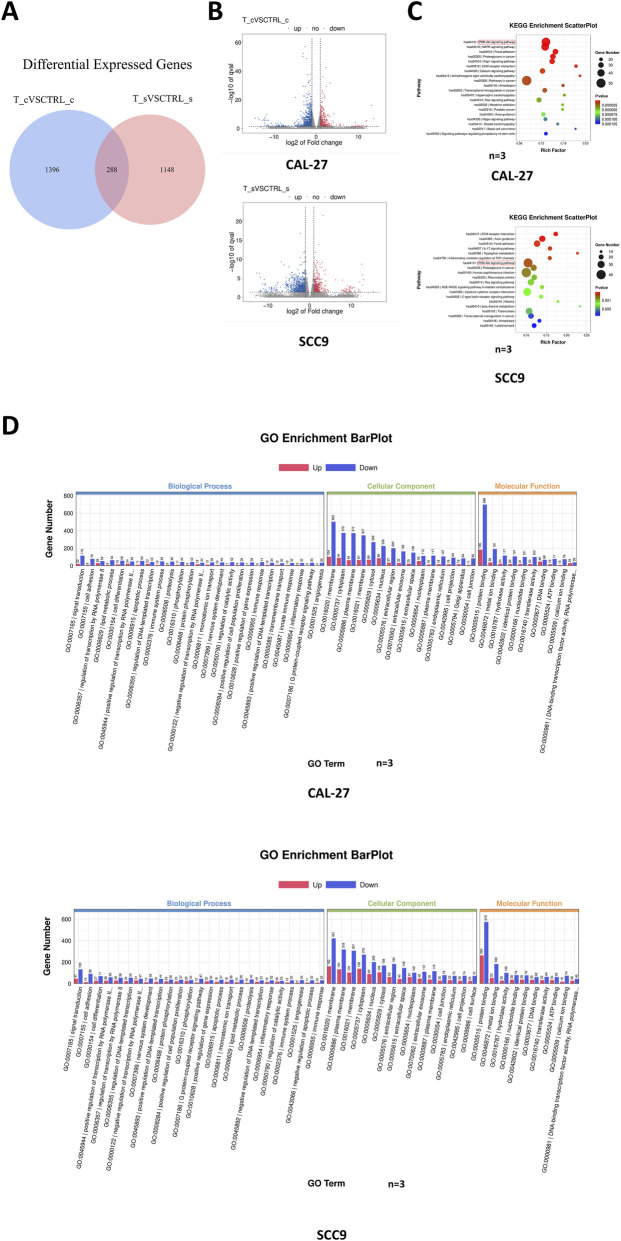
**(A)** Venn diagram of intersection targets activated by **g2** after it acted on Cal-27 and SCC9 cells. The **g2**-treated and control groups of CAL-27 and SCC9 cells share 288 differentially expressed genes (DEGs). **(B)** Transcriptomics analysis was employed to elucidate the molecular mechanisms underlying the therapeutic effects of **g2** against tongue squamous cell carcinoma. A comprehensive comparison of gene expression profiles of the control group and the **g2**-treated group was conducted, with DEGs visualized using a volcano plot. **(C,D)** Gene ontology and KEGG enrichment analysis of **g2** on key protein pathways related to CAL-27 and SCC9 cells was performed.

Following a 24-h treatment with **g2**, total RNA was extracted from CAL-27 and SCC9 cells using TRIzol® Reagent. The RNA samples were subsequently collected and preserved under frozen conditions in preparation for RNA sequencing analysis. Transcriptomic profiling revealed that **g2** treatment induced widespread transcriptional alterations, with a total of 288 differentially expressed genes identified across both cell lines. Compared to untreated control cells, **g2** treatment significantly upregulated the expression of genes associated with the PI3K/AKT signaling pathway in both CAL-27 and SCC9 cells. In addition to the PI3K-Akt pathway, **g2** also modulated multiple signaling pathways implicated in cancer progression, cell adhesion, and immune regulation, suggesting its potential role as a multi-target regulator (Figure 6).

### 3.6 **g** ^**2**^ can act on TSCC cells through the PI3K/AKT/P53 signaling pathway

**FIGURE 7:**
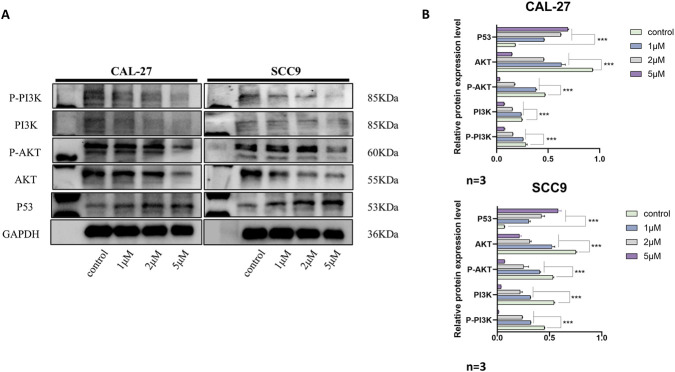
**(A) g2** treatment activated the PI3K/AKT/P53 signaling pathway. CAL-27 and SCC9 cells were treated with **g2** (2 μM) for 24 h. **(B)** Upregulation and downregulation of genes were detected by transcriptome sequencing, and activation of the PI3K/AKT/P53 signaling pathway was identified. ***P < 0.001.

To elucidate the molecular mechanisms underlying the anti-tumor effects of **g2**, its impact on the PI3K/AKT/P53 signaling pathway was investigated using western blotting analysis. The expression levels of five key proteins within this pathway—phosphorylated PI3K (P-PI3K), total PI3K, phosphorylated AKT (P-AKT), total AKT, and the tumor suppressor protein P53—were quantitatively assessed in CAL-27 and SCC-9 cells following treatment with different concentrations of **g2**. The western blotting results revealed that **g2** significantly downregulated the expression of both PI3K and AKT in a dose-dependent manner, suggesting an inhibitory effect on the activation of this oncogenic signaling cascade. Furthermore, a marked upregulation of the pro-apoptotic protein P53 was observed in both cell lines after **g2** treatment, indicating the induction of apoptotic pathways. These findings collectively demonstrate that **g2** exerts its anti-proliferative and pro-apoptotic effects by modulating the PI3K/AKT/P53 signaling pathway in TSCC cells. Specifically, **g2** suppresses the PI3K/AKT axis while concurrently activating P53, thereby inhibiting cell growth and metastasis (Figure 7). The full, uncropped membranes for all biological replicates (n = 5 per group) are provided in Supplementary Figure S3.

### 3.7 Antitumor effect of **g2** *in vivo*

Following *in vitro* investigations, *in vivo* studies were performed utilizing a xenograft model of SSc established by subcutaneously inoculating CAL-27 cells into nude mice. The therapeutic interventions were the administration of **g2** at dosages of 10 and 20 mg/kg. The experimental protocol was initiated when the tumor diameter attained a threshold of 5 mm. By the 25th day, a significant escalation in tumor volume was observed in the untreated control cohort. In contrast, the **g2**-treated groups exhibited a considerable reduction in tumor volume relative to the control group, with the 20 mg/kg group demonstrating a more efficacious suppression of tumor proliferation compared with the 10 mg/kg group. Body weight monitoring revealed no significant reduction in the treatment groups compared with the control group. Histopathological examination of hematoxylin and eosin-stained tissue sections from major organs, including the heart, liver, spleen, lungs, and kidneys, in nude mice showed no evident pathological alterations (Supplementary Figure S1). These findings show the dose-dependent antitumor efficacy of **g2** and lack of systemic toxicity at the tested dosage in this preclinical model, supporting its further development as a potential antitumor agent with a favorable safety profile.

**FIGURE 8:**
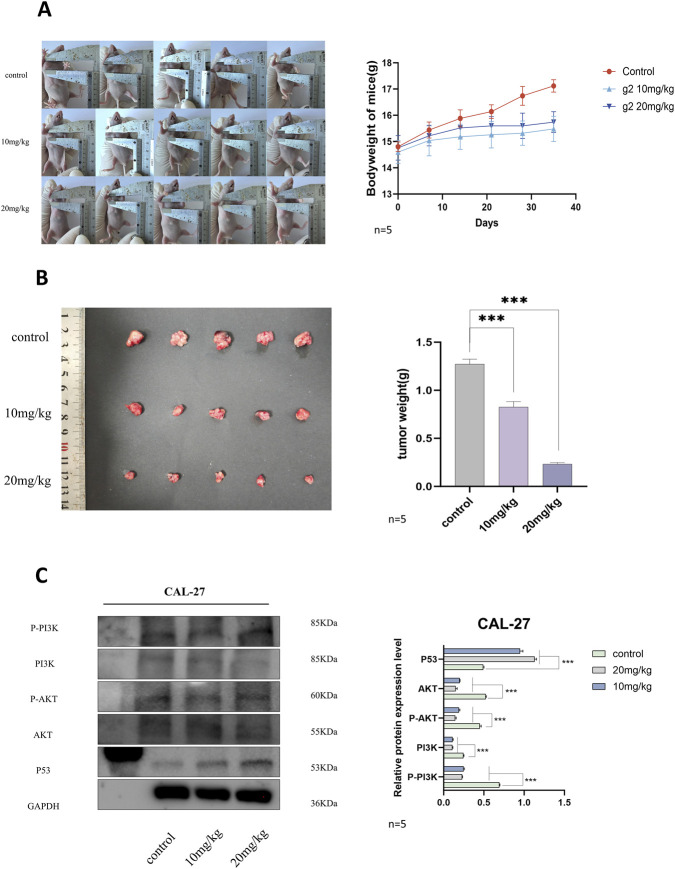
**(A)** After the tumor had grown to 5 mm × 5 mm, mice were left untreated or were treated with **g2** (10 or 20 mg/kg) and euthanized at 35 days. **(B)** After euthanasia, the weight of the tumor was measured, and the treatment and control groups were compared. ***P < 0.001. **(C)** Western blot analysis of tumor tissues demonstrated that **g2** upregulates the expression of the pro-apoptotic protein P53 while downregulating the expression of the anti-apoptotic proteins PI3K and AKT. Dose-dependent effects were observed, with higher doses of **g2** leading to a more pronounced increase in P53 expression and a greater reduction in PI3K and AKT levels compared with the control group. ***P < 0.001.

Immunoblotting analysis of murine tumor tissues revealed dose-dependent alterations in protein expression. At a dosage of 10 mg/kg, a marked upregulation of P53 was observed, concomitant with a reduction in the expression levels of P-PI3K, PI3K, P-AKT, and AKT. Elevating the dose to 20 mg/kg resulted in a further significant increase in P53 expression, while the levels of P-PI3K, PI3K, P-AKT, and AKT exhibited a corresponding decline. These findings indicate a dose-responsive modulation of key signaling pathways associated with tumorigenesis (Figure 8).

## 4 Discussion

Among the various types of cancer, HNSCC represents a major concern (Xie et al., 2024). Within the spectrum of HNSCC, TSCC holds particular significance as the most prevalent form of oral cancer in China. Despite advances in surgical techniques, patients often face substantial post-operative complications and functional impairments (Fang et al., 2025). The prognosis for patients with TSCC remains suboptimal, with the 5-year survival rate hovering below 50% (Zeng et al., 2022). This grim statistic underscores the urgent need for improved therapeutic strategies and early detection methods.

Tryptanthrin derivatives have demonstrated significant antitumor potential across various cancer types (Pinheiro et al., 2022; Zou et al., 2023). Studies reveal that these compounds exhibit inhibitory effects on tumor cell proliferation, migration, and invasion through multiple molecular mechanisms. In hepatocellular carcinoma, tryptanthrin derivatives induce apoptosis by modulating the PI3K/AKT/mTOR pathway (Li et al., 2024). In breast cancer, they suppress tumor growth by regulating the STAT3 signaling cascade (Ma et al., 2020), and in colorectal cancer, they inhibit Wnt/β-catenin signaling. Their ability to target diverse oncogenic pathways underscores their potential as multi-targeted anticancer agents.

This study discovered that the tryptanthrin derivative **g2** may inhibit the activity and metastasis of the TSCC cell lines CAL-27 and SCC9 through the PI3K/AKT/P53 pathway. This pathway is intricately linked to tumorigenesis and significantly influences tumor cell invasion, migration, and apoptosis (Alves and Ditzel, 2023; Wang et al., 2022). Among the most dysregulated pathways in cancer, the PI3K/AKT/P53 pathway is pivotal in driving tumor initiation, progression, and therapeutic resistance (Xu et al., 2020). In patients with advanced cancer, activation of this pathway facilitates autonomous proliferation, inhibits apoptosis, promotes sustained angiogenesis (Chang et al., 2019), enhances tissue invasion and metastatic capabilities, and diminishes sensitivity to growth-inhibitory signals (Yu et al., 2022). These characteristics underscore the pathway’s significance as a promising target for innovative anticancer therapies.

The transcription factor P53, a tumor suppressor that can induce downstream aging or apoptosis-related genes, is often missing or mutated in malignant human tumors (Han et al., 2020). Therefore, the P53 pathway is a target for various anticancer chemotherapy drugs (Liu and Gu, 2022). Research has found that serotonin molecules can activate the P53 pathway to trigger cell cycle arrest and apoptosis in tumor cells (Ghosh et al., 2023; Hassin and Oren, 2022; Marvalim et al., 2023). Although the involvement of this pathway in tumor development has been extensively documented, its specific role and clinical relevance in human TSCC remain insufficiently understood.

*In vitro* studies demonstrate that the tryptanthrin derivative **g2** effectively inhibits the activity of human TSCC cell lines CAL-27 and SCC9. In this study, cell cycle assays revealed significant arrest in TSCC cells treated with **g2**. Western blotting analysis confirmed that **g2** modulates the PI3K/AKT and P53 signaling pathways by downregulating PI3K, AKT, and their phosphorylated forms. Phosphatidylinositol-3 kinases (PI3Ks) are lipid kinases responsible for phosphorylating the 3′-OH group of inositol phospholipids, thereby producing the second messenger phosphatidylinositol 3,4,5-trisphosphate (PIP3) (Deng and Zhou, 2023; Xiao et al., 2022). Upon activation of receptor protein tyrosine kinases, PI3K generates PIP3 and phosphatidylinositol 3,4-bisphosphate at the inner cell membrane. These phospholipids recruit AKT to the intracellular membrane, where it undergoes phosphorylation and activation by PDK1 and PDK2 kinases (Deng and Zhou, 2023; Su et al., 2023). Activated AKT modulates various substrates crucial for cell survival, cell cycle regulation, and growth control (Bu et al., 2024; Sun et al., 2023). Recent research indicates frequent dysregulation of components within the PI3K/AKT/P53 pathway across multiple human cancers (Liu et al., 2024). Cancer therapies, including chemotherapy and radiotherapy, predominantly eliminate malignant cells by inducing apoptosis mechanisms while enhancing P53 acetylation, ultimately inducing apoptosis. These findings suggest that **g2** promotes TSCC cell death via apoptosis-related mechanisms.

*In vivo* experiments utilizing CAL-27 xenograft models in nude mice demonstrated that treatment with the compound **g2** resulted in a significant reduction in tumor volume compared with the control group. At a concentration of 20 mg/kg, **g2** markedly inhibited tumor growth without inducing systemic toxicity or significant body weight loss in nude mice, thereby supporting the compound’s safety profile. Immunohistochemical analysis of tumor tissues revealed that **g2** downregulated the expression of PI3K, P-PI3K, AKT, and P-AKT while upregulating the expression of P53 protein, consistent with the trends observed in the *in vitro* experiments. These findings underscore the potential of **g2** as a therapeutic agent for TSCC and highlight the need for further investigation into its mechanisms and efficacy across diverse cellular contexts.

Despite these promising findings, several limitations of this study should be acknowledged. We acknowledge that this study has limitations. First, while we have demonstrated that **g2** exhibits cytotoxic effects against CAL-27 and SCC9 oral cancer cells, its selectivity toward cancer cells versus normal cells has not been directly assessed. The therapeutic window of **g2** remains to be established through comparative studies using normal oral keratinocytes or fibroblasts. Such experiments are essential to determine whether **g2** selectively targets cancer cells or exerts non-specific cytotoxicity. Future studies will also need to evaluate the safety profile of **g2** *in vivo* using appropriate animal models to assess potential toxicity to vital organs. Secondly, apoptosis was assessed solely by Annexin V-FITC/PI staining followed by flow cytometry, which, although a widely accepted quantitative method, does not provide morphological evidence of apoptosis. Future studies incorporating complementary assays such as AO/EB staining and Western blot analysis of apoptosis-related proteins (e.g., cleaved caspase-3, BAX, BCL2) would further substantiate our findings. Thirdly, our data demonstrate that **g2** inhibits PI3K/AKT phosphorylation and upregulates P53 expression, accompanied by suppressed proliferation/migration and induced apoptosis. This inverse relationship between PI3K/AKT activity and P53 expression is consistent with the established regulatory mechanism wherein AKT phosphorylates MDM2, promoting MDM2-mediated P53 degradation and thereby suppressing P53 activity. However, we acknowledge that direct experimental validation using PI3K/AKT-specific inhibitors/activators or P53 modulation is needed to definitively establish causality. Future studies employing pharmacological inhibitors and genetic approaches (siRNA, overexpression) will be necessary to confirm the hierarchical relationship and its functional consequences. In addition, while compound **g2** demonstrated superior anti-tumor activity compared to other analogues, the structural basis for this enhanced potency remains to be fully elucidated. Although qualitative structural comparisons suggest that the substitution position, linker length, and terminal amino group may contribute to activity, quantitative structure-activity relationship (SAR) analysis and molecular docking studies are needed to provide mechanistic insights. Future studies in collaboration with computational chemistry groups will aim to perform molecular docking to predict the binding mode of **g2** with potential targets such as Bcl-2, and to systematically explore SAR through synthesis of additional analogues. These investigations will help validate the proposed mechanisms and guide further optimization of this promising compound class. Finally, animal models, including subcutaneous and orthotopic xenografts using genetically modified cells, are essential to confirm the anti-tumor and anti-metastatic effects observed *in vitro* and to assess potential toxicity. Future studies employing nude mice are warranted to evaluate the therapeutic potential of **g2** in a more physiologically relevant context. Additionally, clinical samples or patient-derived xenografts (PDXs) would further strengthen the translational relevance of our findings.

## 5 Conclusion

In summary, the findings of this study demonstrate that the tryptanthrin derivative **g2** exhibits consistent antitumor effects on CAL-27 and SCC9 cells through multiple mechanisms, including inhibition of cell migration and invasion, induction of apoptosis, and cell cycle arrest. Specifically, they revealed that **g2** modulates the PI3K/AKT/P53 signaling pathway by downregulating PI3K and AKT while upregulating P53 and its phosphorylated forms, thereby activating apoptotic mechanisms to eliminate malignant cells. Taken together, the *in vitro* and *in vivo* experimental data identify **g2** as a promising therapeutic candidate for TSCC, suggesting its potential as a novel chemotherapeutic agent and providing preliminary evidence supporting its future clinical development.

## Data Availability

The raw gene count matrices generated from RNA-seq transcriptome sequencing in this study are publicly available in Figshare. The dataset DOI is https://doi.org/10.6084/m9.figshare.32688201.
