# Supplementary material for: The tryptanthrin derivative g2 suppresses tongue squamous cell carcinoma via PI3K/AKT/P53 cathway modulation: evidence from in vitro and in vivo studies
Source: Front Pharmacol. 2026 Jun 18;17:1789002. doi: 10.3389/fphar.2026.1789002 (PMC13323819; doi:10.3389/fphar.2026.1789002)
Supplement: Supplementary file 1 [file DataSheet1.docx]

***Contents***

[**Supplementary Table 1.** 3](#_Toc230442000)

[**Supplementary Figure 1.** 4](#_Toc230442001)

[5](#_Toc230442002)

[**Supplementary Figure 2.**. 5](#_Toc230442003)

[6](#_Toc230442004)

[**Supplementary Figure S3.** 7](#_Toc230442005)

Determination of LD_50_ of **g2**

CAL-27 cells (5 × 10^7^) were suspended in 100 μL of sterile PBS. Twenty nude mice were randomly divided into five groups, **g2** were injected into the right axilla of nude mice. According to the results of the acute toxicity test, LD_50_=81.549 (72.893-101.069) mg/Kg (9.5% confidence interval, **Table.S1**), we chose injection doses of 10 and 20 mg/Kg.

**Supplementary Table 1.** LD_50_ determination results of compound **g2** on mice after intragastric administration for 7 days

| Group | Dose (mg/kg) | Number of deaths | Mortality rate (%) |
| --- | --- | --- | --- |
| 1 | 30 | 0 | 0 |
| 2 | 45 | 0 | 0 |
| 3 | 60 | 1 | 10 |
| 4 | 75 | 3 | 30 |
| 5 | 90 | 7 | 70 |

LD50=81.549（72.893-101.069）mg/Kg

**Safety**

The safety profile of compound **g2** as a potential anti-tumor agent was rigorously evaluated. No significant changes in body weight were observed in nude mice throughout the treatment period. Histopathological analysis via hematoxylin and eosin (H&E) staining revealed preserved tissue architecture in the heart, liver, spleen, lungs, and kidneys, with no evidence of drug-induced toxicity or metastatic lesions (**Figure S1**). These findings support the preliminary safety of **g2** for further therapeutic development.
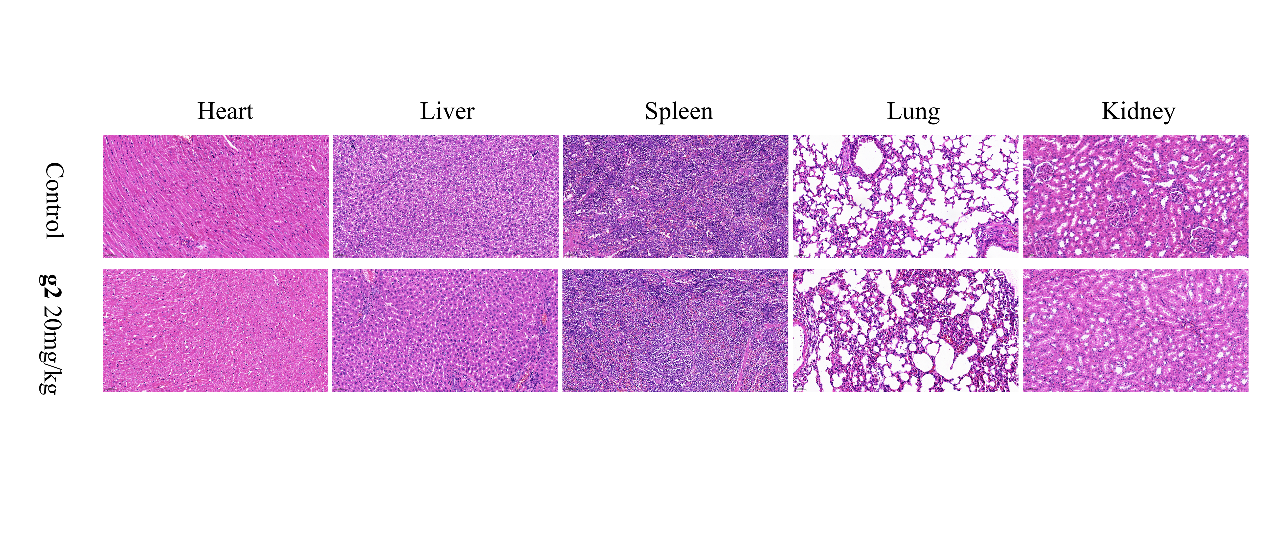


**Supplementary Figure 1.** HE-stained tissue sections of nude mouse organs.


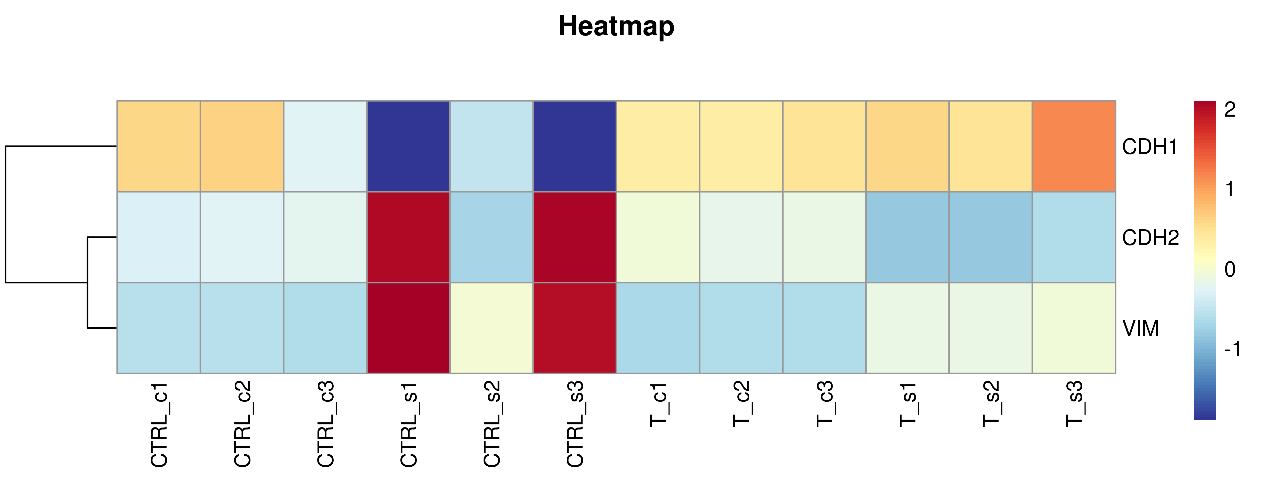


**Supplementary Figure 2.** Expression of EMT-related genes in CAL-27 and SCC9 cells. Heatmap showing the mRNA expression levels of CDH1 (E-cadherin), VIM (Vimentin), and CDH2 (N-cadherin) in control and **g2**-treated cells. Color scale represents relative expression levels. Treatment with **g2** upregulated CDH1 expression, while VIM and CDH2 showed minimal changes.


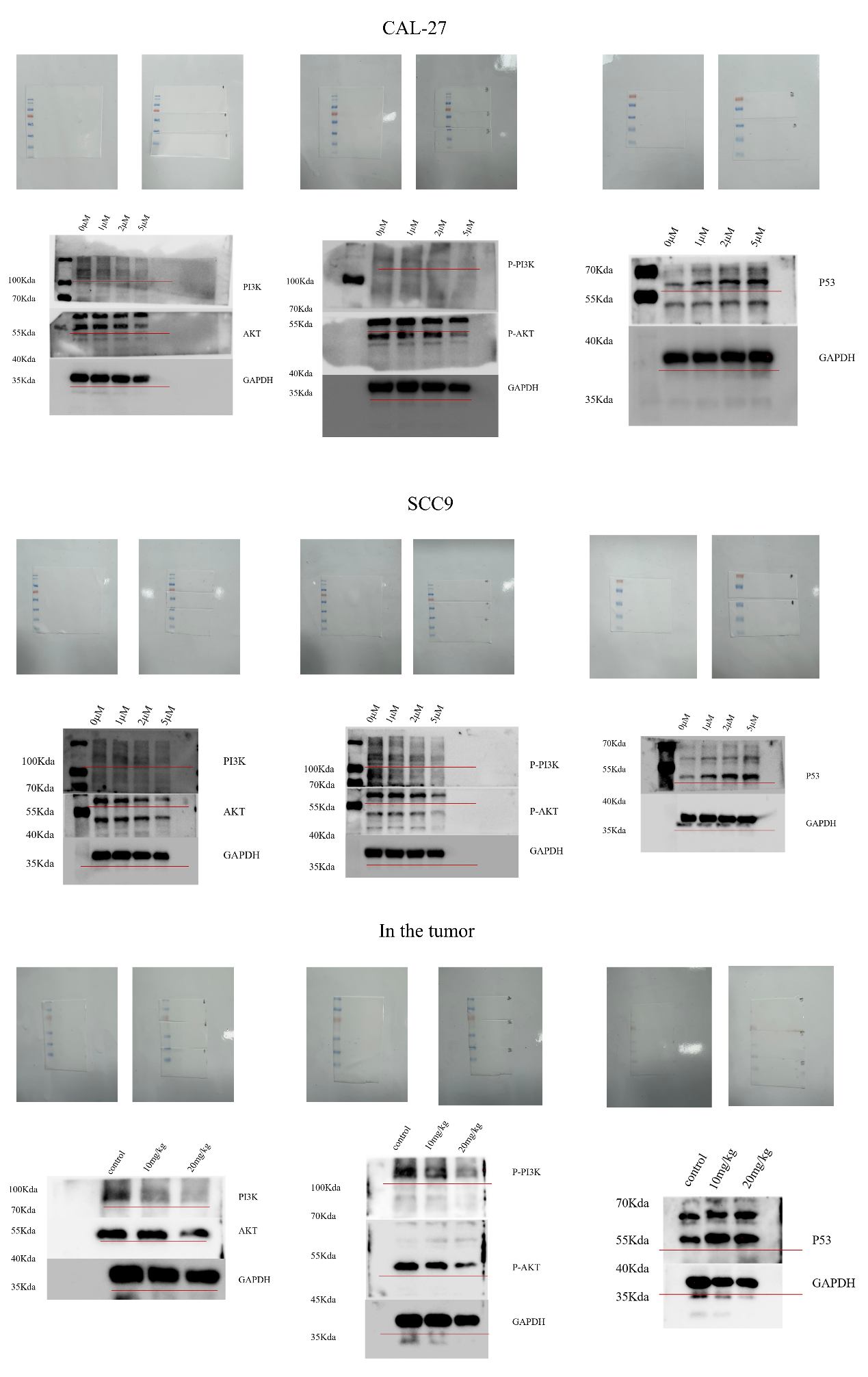


**Supplementary Figure 3.** Uncropped full Western blot membranes for **Figure 7** and **Figure 8**.
